# Effects of the COVID-19 pandemic on Czech citizens: how do depression and anxiety symptoms influence cognitive, behavioral, and emotional changes?

**DOI:** 10.3389/fpsyg.2023.1204824

**Published:** 2023-06-26

**Authors:** Dagmar Hajkova, Jan Sandora, Radka Žídková, Klara Malinakova, Lukas Novak

**Affiliations:** Olomouc University Social Health Institute, Palacký University in Olomouc, Olomouc, Czechia

**Keywords:** depression, anxiety, alcohol, tobacco, food consumer behavior

## Abstract

**Background:**

This study examined the impact of anxiety and depression symptoms during the first stage of the COVID-19 pandemic on the behavioral, cognitive, and emotional changes of the Czech population.

**Methods:**

The research sample (*n* = 2363; 48.83 ± 16.53 years; 50.15% men) was obtained using an online survey. Depression and anxiety symptoms were measured using the Overall Depression Severity and Impairment Scale (ODSIS) and the Overall Anxiety Severity and Impairment Scale (OASIS) and associations were adjusted for age, gender, and economic status.

**Results:**

The results showed that increased symptoms of anxiety and depression were significantly linked to feelings of loneliness, helplessness, reduced quality of relationship with a partner, higher probabilities of alcohol abuse, food consumption, and contemplation of existential questions. Higher symptoms of anxiety were associated with feelings of being threatened. Higher symptoms of depression symptoms increased tobacco abuse.

**Conclusion:**

During the first stage of the COVID-19 pandemic, higher symptoms of anxiety and depression among Czech citizens were associated with behavioral, cognitive, and emotional changes.

## Introduction

During the first stage of the COVID-19 pandemic, the prevalence of depression and anxiety significantly increased worldwide (Xiong et al., 2020; Pieh et al., 2021; Fairlamb, 2022; Yu et al., 2022). The United Nations has issued a warning regarding the emergence of mental health crisis related to COVID-19 (Xiong et al., 2020). It is essential to highlight that this crisis continues to persist even after the pandemic (Ren and Guo, 2020). Several researchers have documented the deleterious impact of the pandemic on mental health, particularly in terms of the psychological distress caused by fear of infection, social isolation, and uncertainty about the future (Tudehope et al., 2022; Diotaiuti et al., 2023). For example, research conducted in China revealed a higher incidence of depression and anxiety symptoms among the general population during the first stage of the pandemic (Wang et al., 2020). Similarly, a study in the United States reported a significant increase in the prevalence of depression and anxiety in response to the pandemic (McGinty et al., 2020). Moreover, in Italy, a country that experienced a severe outbreak of COVID-19, high levels of depression and anxiety symptoms were reported among the population (Diotaiuti et al., 2021, 2023). During the first wave of the COVID-19 pandemic, the Czech Republic was among the countries in Europe which was most influenced and a significant increase in anxiety and depressive symptoms was observed among the general population, with a prevalence of 15.5% and 12.5% (Trnka and Lorencova, 2020).

Individuals experiencing symptoms of depression and anxiety often report experiencing a range of emotional, behavioral, and cognitive changes that can significantly impact their daily lives. The first stage of the pandemic was marked by a variety of emotional changes, including feelings of fear, anger, and hopelessness (Trnka and Lorencova, 2020; Zidkova et al., 2021), which can lead to depression and anxiety symptoms. Behavioral changes related to depression and anxiety symptoms have been correlated with a higher incidence of alcohol and tobacco abuse (Clay and Parker, 2020; Xiong et al., 2020; Bountress et al., 2022). Furthermore, other studies have shown that certain individuals who have not modified their lifestyle habits during the pandemic, such as physical activity during the lockdown, may be at an elevated risk of developing emotional eating patterns or engaging in more frequent food consumption (D'Oliveira et al., 2022; Ferrara et al., 2022; Galli et al., 2022; Pak et al., 2022). Cognitive changes involving contemplation of existential questions, prayer, and matters of religion emerged among individuals during the COVID-19 pandemic (Killgore et al., 2020; Tomaszek and Muchacka-Cymerman, 2020).

Taken together, there are many pieces of research on the topic COVID-19 pandemic related to behavioral changes (Chan et al., 2021; Fu et al., 2021; Lee et al., 2021), emotional changes (Fu et al., 2021; Chen et al., 2022; Dominte et al., 2022), and cognitive changes (Thagard, 2021), but none of the research examines all three types of changes at once relating to anxiety and depression symptoms in the Czech Republic. The finding emphasizes an urgent need to scale up to more researchers in this area. This article aims to find out how the first stage of the COVID-19 pandemic manifested behavioral, emotional, and cognitive changes relating to depression and anxiety symptoms.

## Methods

### Participants and procedure

We collected data in two stages on a national sample of the Czech population aged between 18 and 97 years. The dataset was collected between April 2020 and June 2020. The first sample is from the first stage of COVID-19 among adults (*n* = 1393) between April 2020 and the beginning of May 2020. The second sample is also from the first stage of COVID-19 among adults (*n* = 972) from the end of May 2020 to June 2020; an online survey was used for both models. Researchers from Olomouc University Social Health Institute designed the study, and the survey was distributed by a professional agency, which also collected the data. Thanks to this process, the sample is balanced in terms of gender, age, and education and has almost representative characteristics.

The dataset from two samples included 2,365 participants, and 2 were excluded because of low-quality data (short response time and a unified pattern of responses). This led to the final sample of 2,363 participants (mean age = 48.83, SD = 16.53, 50.15% men). Participation in the survey was anonymous and voluntary. Participants were allowed to leave the study at any time without stating a reason for their decision.

### Measures

**Emotional changes** focused on anxiety and depression symptoms were measured by questionnaires, including the Overall Anxiety Severity and Impairment Scale (OASIS) for anxiety symptoms and the Overall Depression Severity and Impairment Scale (ODSIS) for depression symptoms.

### Overall anxiety severity and impairment scale

The Overall Anxiety Severity and Impairment Scale (OASIS) is a self-report questionnaire used to measure the severity and functional impact of anxiety symptoms. It consists of five items that assess the frequency, intensity, and interference of anxiety symptoms with daily life activities, such as work and social interaction. Participants chose one of five responses, ranging from 0 (never) to 4 (all time), which best illustrated their experience of the symptoms over the past week. The final score ranges from 0 to 20; higher scores mean more significant problems with anxiety. The OASIS has been shown to have good internal consistency, test–retest reliability, and convergent validity with other measures of anxiety. It is a widely used measure in both clinical and research settings for assessing anxiety symptoms severity and treatment outcome (Norman et al., 2006; Campbell-Sills et al., 2009). We used the Czech version of the abbreviated Overall Anxiety Severity and Impairment Scale (OASIS) validated by Sandora et al. (2021). The cutoff score in the Czech version of OASIS was identified as 15 (Sandora et al., 2021).

### Overall depression severity and impairment scale

The Overall Depression Severity and Impairment Scale (ODSIS) is a self-reported questionnaire used to assess the severity of depression and its impact on daily functioning. The ODSIS measures both the cognitive and affective aspects of depression, including symptoms such as sadness, guilt, and hopelessness, as well as functional impairment in areas such as work, school, and relationships (Kotov et al., 2010). The questionnaire consists of nine items with responses rated on a 5-point scale. The ODSIS is a reliable and valid measure of depression severity and impairment in both clinical and non-clinical populations (Kotov et al., 2010). We used the Czech version of the abbreviated Overall Depression Severity and Impairment Scale (ODSIS) validated by Sandora et al. (2021). The final score ranges from 0 to 20; higher scores mean more significant problems with depression. The cutoff score for the original version was defined as ≥8. Different countries, cultures, and samples of participants have other cutoff values, ranging from 5 to 12. The cutoff score in the Czech version of ODSIS was identified as 12 (Sandora et al., 2021).

### Behavioral and cognitive changes

The study also measured behavioral and cognitive changes by asking participants whether there were any changes in their daily activities in various areas. These areas were measured by the question: “Has anything changed in your life in the following areas?” with possible answers: (1) “I do this activity less often”, (2) “Unchanged”, and (3) “I do this activity more often”. The researched areas concerned the following: (1) “Thinking about existential questions”, (2) “Thinking about religion”, (3) “Prayer”, (4) “Drinking alcohol”, (5) “Eating food”, (6) “Smoking or chewing tobacco”, (7) “Shopping for new things”, and others. Participants were asked to choose from three possible responses: doing the activity less often, unchanged, or more often. The responses were later dichotomized into two groups for further analysis: “less often/unchanged” and “more often”. The full list of areas and their corresponding responses can be found in **Table 4**. This method provided insight into how individuals coped with the pandemic and any associated stressors in the first stage of COVID-19.

### Statistical analysis

First, we determined the sociodemographic characteristics of the background characteristics of the sample and examined the data distribution using Mardia's test of skewness and kurtosis, which suggested slight heteroscedasticity. Our data were not normally distributed. As a result, we used Spearman rank correlation and non-parametric group comparison tests to explore relationships between variables of interest. The effect size for the non-parametric group comparison was monitored by the Dunn and Gamas–Howell tests. Furthermore, we used the Bonferroni correction.

Second, we assessed the association of anxiety and depression in COVID-19 pandemic-related changes in emotions and relationships using two logistic regression models, one for anxiety (measured by OASIS) and the second for depression (measured by ODSIS) adjusted for gender, age, and socioeconomic status. Each independent variable was assessed in a separate model.

In the same way, we assessed the associations of behavioral and cognitive changes related to the COVID-19 pandemic with OASIS and ODSIS using a binary logistic model.

## Results

The sociodemographic characteristics of the sample are presented in Table 1. The sample of 2363 (mean age = 48.83, SD = 16.53, 50.15% men) was relatively balanced in terms of gender. Higher anxiety measured by OASIS was observed in students than in employed/entrepreneurs and pensioners. Higher anxiety was also noted in religious participants than in non-religious participants. Furthermore, atheists were found to have a significantly lower level of anxiety than religious participants. Higher depression measured by ODSIS was also observed in students than in employed/entrepreneurs and pensioners. Higher depression was also reported in participants who were religious but not members of a church than non-religious.

**Table 1:** Sociodemographic results of the two samples.

**Variable**	**Sample 1**	**Sample 2**	**OASIS differences**	**ODSIS differences**
**Gender**				
1. Male	483 (50%)	702 (50%)		
2. Female	487 (50%)	691 (50%)		
**Family status**				
1. No relationship	496 (51%)	476 (34%)		
2. In the relationship	474 (49%)	917 (66%)		
**Education**				
1. Elementary school	86 (8.9%)	113 (8.1%)		
2. Secondary vocational school	367 (38%)	636 (46%)		
3. Secondary school with graduation	354 (37%)	467 (34%)		
4. College/University	124 (13%)	177 (13%)		
**Economic status**				
1. Employed/Entrepreneur	526 (54.4%)	754 (54%)		
2. In household/without work	88 (9.1%)	114 (8.9%)		
3. Pensioner	300 (31%)	438 (31%)	5 > 4 (*p =* 0.008, Â = 0.35)	5 > 4 (*p =* 0.010, Â = 0.35)
4. Student	52 (5.4%)	77 (5.5%)	5 > 6 (*p =* 0.008, Â = 0.36)	5 > 6 (*p =* 0.011, Â = 0.36)
**Religiosity**				
1. Convinced atheist		179 (13%)	1 < 4 (*p =* 0.014; Â = 0.4), 1 < 3 (*p =* 0.031; Â =0.57),	
2. Non-believer		731 (52%)	3 > 2 (*p =* 0.001, Â = 0.43)	3 > 2 (*p =* 0.006, Â = 0.42)
3. Believer outside the church		362 (26%)	5 > 6 (*p =* 0.002, Â = 0.4)	
4. Believer, member of the church		121 (8.7%)		

A, Vargha and Delaney's A effect size, sociodemographic differences among more than two groups were calculated using the Dunn test, and two group comparison was conducted using the Wilcoxon rank-sum test; r, Wilcoxon rank-sum test effect size; HSPS.T, Highly Sensitive Person Scale–total score; AES, esthetic sensitivity subscale; EOE, ease of excitation subscale.

In Table 2, logistic regression indicated that with increasing depression (measured by OASIS) during the current COVID-19 pandemic, there was a general decline in the quality of relationships, especially in relationships with a partner, by 16.82% (*p* < 0.001). A significant association between the deterioration of emotions during the COVID-19 pandemic and OASIS has also been found: with one-unit increase in the OASIS score, the probability of relapse of loneliness increased by 18.1%, threat by 18.15%, and helplessness by 22.38%.

**Table 2:** Relationship (in odds ratios) between anxiety symptoms and the COVID-19 pandemic-related changes in emotions and relationships.

			**Deterioration**						
	**Relationship with the partner**	**Relationship with the children**	**Rel. with others in the household**	**Loneliness**	**Feelings of threat**	**Fear and anxiety**	**Helplessness**	**Hope**	**Day structure**
Crude	1.17^***^ [1.11, 1.23]	1.12^***^ [1.05, 1.19]	1.11^***^ [1.04, 1.18]	1.18^***^ [1.14, 1.22]	1.18^***^ [1.15, 1.22]	1.29^***^ [1.25, 1.34]	1.22^***^ [1.18, 1.27]	1.16^***^ [1.12, 1.21]	1.05^***^ [1.02, 1.08]
Adjusted	1.19^***^ [1.13, 1.25]	1.11^***^ [1.05, 1.18]	1.13^***^ [1.07, 1.20]	1.20^***^ [1.16, 1.24]	1.18^***^ [1.14, 1.21]	1.30^***^ [1.25, 1.35]	1.23^***^ [1.19, 1.27]	1.17^***^ [1.12, 1.22]	1.06^***^ [1.03, 1.09]

^*^p < 0.05, ^**^p < 0.01,

*** *p* < 0.001. The adjusted effect was calculated using the following variables as covariates: age, gender, socioeconomic status, education, religiosity, and neuroticism. After the Bonferroni correction, only results with *p* < 0.001 remained significant (except the adjusted effect on food consumption). Values in brackets refer to the 95% confidence interval of odds ratios.

Similar associations were found in ODSIS in Table 3: a one-unit increase in the ODSIS score was associated with a higher probability of deterioration of relationship quality with a partner by 15.89%, a higher probability of relapse of loneliness by 16.77%, and helplessness by 17.95%. These results are adjusted for age, gender, and economic status.

**Table 3:** Relationship (in odds ratios) between depression symptoms and the COVID-19 pandemic-related changes in emotions and relationships.

			**Deterioration**						
	**Relationship with the partner**	**Relationship with the children**	**Rel. with others in the household**	**Loneliness**	**Feelings of threat**	**Fear and anxiety**	**Helplessness**	**Hope**	**Day structure**
Crude	1.16^***^ [1.10, 1.22]	1.12^***^ [1.06, 1.19]	1.11^***^ [1.05, 1.17]	1.18^***^ [1.14, 1.22]	1.18^***^ [1.15, 1.22]	1.29^***^ [1.25, 1.34]	1.22^***^ [1.18, 1.27]	1.16^***^ [1.12, 1.21]	1.05^***^ [1.02, 1.08]
Adjusted	1.18^***^ [1.12, 1.23]	1.12^***^ [1.06, 1.19]	1.13^***^ [1.07, 1.20]	1.20^***^ [1.16, 1.24]	1.18^***^ [1.14, 1.21]	1.30^***^ [1.25, 1.35]	1.23^***^ [1.19, 1.27]	1.17^***^ [1.12, 1.22]	1.06^***^ [1.03, 1.09]

^*^p < 0.05, ^**^p < 0.01,

*** *p* < 0.001. The adjusted effect was calculated using the following variables as covariates: age, gender, socioeconomic status, education, religiosity, and neuroticism. After the Bonferroni correction, only results with *p* < 0.001 remained significant (except the adjusted effect on food consumption). Values in brackets refer to the 95% confidence interval of odds ratios.

Behavioral and cognitive changes related to the COVID-19 pandemic were significantly associated with OASIS and ODSIS. Specifically, a one-unit increase in the OASIS score resulted in a higher probability of alcohol drinking by 7.84% and increased food consumption by 8.27%. ODSIS was associated with an even higher number of health-linked behaviors: with an increasing score on ODSIS, the probability of more frequent food consumption increased by 7.77%, alcohol drinking by 7.67%, and smoking or chewing tobacco by 8.64%. These results are adjusted for age, gender, and economic status in Table 4.

**Table 4:** Associations of behavioral and cognitive changes related to the COVID-19 pandemic and their relationship with anxiety and depression.

	**OR OASIS**	**OR OASIS na**	**OR ODSIS**	**OR ODSIS na**
Thinking about existential questions	1.13^***^ [1.09, 1.16]	1.12^***^ [1.09, 1.16]	1.10^***^ [1.07, 1.13]	1.10^***^ [1.07, 1.13]
Thinking about religion	1.10^***^ [1.04, 1.16]	1.11^***^ [1.05, 1.16]	1.10^***^ [1.04, 1.15]	1.10^***^ [1.05, 1.15]
Prayer	1.09^***^ [1.04, 1.15]	1.10^***^ [1.05, 1.15]	1.09^***^ [1.04, 1.14]	1.09^***^ [1.04, 1.14]
Smoking or chewing tobacco	1.07^**^ [1.02, 1.12]	1.08^***^ [1.04, 1.13]	1.09^***^ [1.04, 1.13]	1.11^***^ [1.06, 1.15]
Alcohol drinking	1.08^***^ [1.03, 1.13]	1.10^***^ [1.06, 1.15]	1.08^***^ [1.03, 1.12]	1.11^***^ [1.06, 1.15]
Buying new things	1.05 [0.98, 1.11]	1.07^*^ [1.01, 1.14]	1.03 [0.97, 1.09]	1.06 [1.00, 1.12]
Food consumption	1.08^***^ [1.05, 1.12]	1.11^***^ [1.07, 1.14]	1.08^***^ [1.04, 1.11]	1.10^***^ [1.07, 1.14]
Sex	1.01 [0.96, 1.06]	1.04 [0.99, 1.09]	1.02 [0.97, 1.07]	1.06^*^ [1.01, 1.10]
Physical activity	0.98 [0.94, 1.01]	1.00 [0.97, 1.04]	0.99 [0.95, 1.02]	1.01 [0.98, 1.05]
Reading	1.02 [0.99, 1.05]	1.03^*^ [1.00, 1.06]	1.01 [0.98, 1.04]	1.02 [0.99, 1.05]
Self-education	1.01 [0.97, 1.05]	1.03 [0.99, 1.07]	1.00 [0.96, 1.04]	1.02 [0.99, 1.06]
Work	1.02 [0.99, 1.06]	1.04^*^ [1.00, 1.08]	1.01 [0.98, 1.05]	1.03 [1.00, 1.07]
Calls	1.03^*^ [1.00, 1.06]	1.04^**^ [1.02, 1.07]	1.02 [0.99, 1.05]	1.02 [1.00, 1.05]
Other forms of online communication	1.06^***^ [1.03, 1.09]	1.08^***^ [1.05, 1.11]	1.04^**^ [1.02, 1.07]	1.06^***^ [1.04, 1.09]

* *p* < 0.05,

** *p* < 0.01,

*** *p* < 0.001. OR, adjusted odds ratios for age, gender, and economical status; na, non-adjusted odds ratios. Only results with *p* < 0.001 survived Bonferroni correction, values in squared brackets refer to the 95% confidence interval of odds ratios.

## Discussion

This study is the first attempt to investigate the impact of anxiety and depression symptoms on the behavioral, cognitive, and emotional changes experienced by the Czech population during the first stage of the COVID-19 pandemic. Our findings reveal that negative emotions such as loneliness and helplessness were significantly associated with higher levels of anxiety and depression symptoms. Furthermore, perceiving a sense of threat was related to increased levels of anxiety symptoms. We also found that the deterioration of a relationship with a partner was linked to higher levels of anxiety and depression symptoms. Additionally, we discovered that levels of anxiety and depression were significantly related to a greater probability of alcohol abuse and heightened food consumption. Moreover, individuals with higher levels of depression symptoms were also more likely to engage in smoking or chewing tobacco. Interestingly, we observed that individuals with higher levels of depression symptoms were more likely to engage in prayer and contemplate existential questions related to religion.

We found that individuals with depression and anxiety symptoms experienced emotional changes such as feelings of loneliness and helplessness, while those with just anxiety symptoms reported a sense of threat. These emotional changes due to COVID-19 occurred all over the world (Wang et al., 2020; Xiong et al., 2020), for example, research conducted in Spain found that the lockdown had significant emotional changes for individuals with depression and anxiety symptoms and stress, which were observed in 20% to 30% of participants. Psychological stress was found in 47.5% of participants (Odriozola-González et al., 2022). The restriction of physical activity and disruption of daily routines may have disrupted people's ability to cope with stressors, further exacerbating symptoms of depression and anxiety. For instance, findings from Italy have demonstrated the effectiveness of a 4-week-based physical exercise protocol for older people in social isolation in improving psychological variables such as anxiety, mood, depression, and stress. This intervention proved to help reduce anxiety and depression symptoms (D'Oliveira et al., 2022). Predictors of physical activity are autonomous motivation and intention. The direct effect of autonomous motivation on physical activity is stronger in participants with low anxiety (Galli et al., 2022). Furthermore, we found out that feeling threatened is linked to anxiety symptoms. Individuals with anxiety tend to experience a heightened sense of threat, which may manifest as a preoccupation with potential danger and an exaggerated fear response. The pandemic situation in the first stage of COVID-19 triggered anxiety symptoms much more than fear (Coelho et al., 2020). In contrast, individuals with depression symptoms may be too exhausted or demotivated to perceive threats in their environment.

According to our study, individuals who suffer from both symptoms of anxiety and depression are prone to alcohol abuse and higher food consumption. The Czech Republic ranks among Europe's nations with the highest alcohol consumption (Svačinová et al., 2022). There is a significant association between perceived stress and changes in alcohol consumption (Tudehope et al., 2022) and also a relationship between reward sensitivity and coping in determining alcohol use (Feil and Hasking, 2008). In the context of our research, increased alcohol consumption associated with anxiety and depression symptoms probably occurred precisely because of overwhelming stress in the first stage of COVID-19, high isolation, and forced public avoidance. However, alcohol was not the only avoidance strategy. For instance, a study conducted on Italian adolescents during the first COVID-19 lockdown found that impulsivity and depressive brooding were predictive factors for internet addiction, with 28% of participants in the full dependency range and 34.7% demonstrating internet abuse behavior (Diotaiuti et al., 2022). Higher food consumption has been suggested as another potential underlying mechanism of coronavirus-related stress and may be utilized as a coping strategy for emotional regulation (Wang et al., 2020). Conversely, those individuals who maintain a balanced, positive mindset and focused on the past and future were found to be less likely to exhibit symptoms of food addiction (Bracale and Vaccaro, 2020; Borisenkov et al., n.d.).

Additionally, we found that individuals with depression symptoms tend to make behavioral changes toward tobacco abuse. This result is consistent with previous research that has demonstrated a positive association between depression and smoking (Rigotti et al., 2021; Chen et al., 2022). Our study contributes to the growing body of evidence that suggests a potential link between coping strategies for individuals with depression symptoms and behavior changes in tobacco abuse.

Finally, the study showed cognitive changes such as interest in more frequent prayer, thinking about religion, and existential questions. It is interesting because the Czech Republic is one of the most atheist countries in the Christian world (Furstova et al., 2021). The global outbreak of COVID-19 has set new challenges for every human being, and it can be compared to a profound existential crisis or a traumatic experience (Tomaszek and Muchacka-Cymerman, 2020). The experience of existential anxiety during the pandemic has led many individuals to become more cognizant of their mortality and to increase their frequency of prayer (Killgore et al., 2020; Tomaszek and Muchacka-Cymerman, 2020). People's sources of meaning in life help to achieve psychological adaptation (Chen et al., 2022). Further research findings suggest that heightened existential questions may be a neglected factor in increasing depression during the pandemic (Fairlamb, 2022). Our findings of cognitive changes can be challenging for the atheist state of the Czech Republic.

We found that symptoms of depression and anxiety contributed to the deterioration of relationships with a partner during the first stage of the COVID-19 pandemic. Behavioral, emotional, and cognitive changes may have led to more conflicts and decreased communication between partners.

### Limitations and strengths

While our study provides valuable insights, it is important to acknowledge its limitations. First, the data collection was conducted during the COVID-19 pandemic, which could have affected the number of responses and the willingness of participants to take part in the study, potentially influencing the results. Second, the use of self-reported measures and short questionnaires may introduce information bias, as participants' responses can be influenced by their current mood.

The study has several important strengths; the most essential is the large study sample of Czech adults during the first stage of COVID-19, which at the time of the survey was the worst-affected country in Europe and the third-worst affected country in the world. It is the first research that examined behavioral, emotional, and cognitive changes related to depression and anxiety during this period in the Czech Republic. The model was close to the nationally representative sample because of its characteristics. It was well-balanced regarding the gender and age of the respondents. Moreover, the data used in the study were cross-sectional. The study showed the situation during the first stage of COVID-19 in the Czech environment.

### Implications of the study

Our study reveals that the initial phase of the COVID-19 pandemic had a profound impact on the Czech population, as evidenced by a surge in emotional, cognitive, and behavioral changes due to symptoms of depression and anxiety. Higher symptoms of depression and anxiety were associated with behavioral changes such as higher levels of food consumption, alcohol abuse, and the deterioration of relationships with a partner. In addition, our findings indicate that symptoms of depression were significantly linked to tobacco abuse. Notably, the pandemic also brought about cognitive changes, with an increased interest in prayer, religion, and existential questions. All these changes pose new challenges for the Czech citizenry.

These findings show a greater need for primary and selective mental health prevention focused on healthy stress-coping strategies related to depression and anxiety instead of alcohol abuse, tobacco abuse, and increased food consumption. One option provides prevention programs on positive stress-coping strategies and self-care.

Future studies can focus more on cognitive, behavioral, and emotional changes after COVID-19 related to anxiety and depression symptoms to find out how the situation continues in the Czech Republic.

## Conclusion

During the first stage of COVID-19 and the subsequent implementation of restrictive measures such as lockdowns, symptoms of depression and anxiety increased among residents of the Czech Republic. These symptoms were associated with various emotional, behavioral, and cognitive changes. Individuals with depression and anxiety symptoms experienced emotional changes, such as feelings of hopelessness and loneliness, while those with anxiety symptoms reported a heightened sense of threat. Behaviorally, individuals with depression and anxiety symptoms exhibited higher levels of alcohol consumption and increased food intake, leading to strain in their relationships with partners. Conversely, those with depressive symptoms alone exhibited tobacco abuse. Cognitively, participants reported an increased interest in existential questions, faith, and prayer, which is noteworthy given the Czech Republic's status as one of the most atheistic countries in the world.

Considering the great influence that symptoms of depression and anxiety appeared to have on Czech citizens' behavioral, emotional, and cognitive changes, the primary and selective mental health prevention focus on healthy stress-coping strategies among people to achieve common goals should be promoted and supported.

To decrease the impact of the pandemic, individuals must take personal responsibility for detecting early symptoms of depression and anxiety and addressing them with positive coping strategies, thus affecting emotional, behavioral, and cognitive changes. To encourage individuals to take responsibility, an effective and accessible tool is necessary to help them detect symptoms, such as the short questionnaires OASIS and ODSIS.

## Data availability statement

The original contributions presented in the study are included in the article/supplementary material, further inquiries can be directed to the corresponding author.

## Ethics statement

The studies involving human participants were reviewed and approved by Ethics Committee of the Faculty of Theology of Palacky University in Olomouc, Czech Republic (No. 2020/06). The patients/participants provided their written informed consent to participate in this study. Written informed consent was obtained from the individual(s) for the publication of any potentially identifiable images or data included in this article.

## Author contributions

All authors listed have made a substantial, direct, and intellectual contribution to the work and approved it for publication.
