# Corrigendum: Effects of the COVID-19 pandemic on Czech citizens: how do depression and anxiety symptoms influence cognitive, behavioral, and emotional changes?

**DOI:** 10.3389/fpsyg.2024.1386166

**Published:** 2024-03-06

**Authors:** Dagmar Hajkova, Jan Sandora, Radka Žídková, Klara Malinakova, Lukas Novak

**Affiliations:** Olomouc University Social Health Institute, Palacký University in Olomouc, Olomouc, Czechia

**Keywords:** depression, anxiety, alcohol, tobacco, food consumer behavior

In the published article, the Funding information was omitted. The correct Funding statement is shown below.

